# Everyday uses of standardized test information in a geriatric setting: a qualitative study exploring occupational therapist and physiotherapist test administrators’ justifications

**DOI:** 10.1186/1472-6963-14-72

**Published:** 2014-02-17

**Authors:** Kariann Krohne, Sandra Torres, Åshild Slettebø, Astrid Bergland

**Affiliations:** 1 Faculty of Health Sciences, Oslo and Akershus University College of Applied Sciences, Oslo 0130, Norway; 2 Department of Sociology, Uppsala University, Uppsala 751 26, Sweden; 3 Department of Health and Nursing Science, University of Agder, Grimstad 4879, Norway

**Keywords:** Standardized testing, Physiotherapist, Occupational therapist, Professional practice, Information use, Geriatric patients, Qualitative research, Fieldwork, Interviews

## Abstract

**Background:**

Health professionals are required to collect data from standardized tests when assessing older patients’ functional ability. Such data provide quantifiable documentation on health outcomes. Little is known, however, about how physiotherapists and occupational therapists who administer standardized tests use test information in their daily clinical work. This article aims to investigate how test administrators in a geriatric setting justify the everyday use of standardized test information.

**Methods:**

Qualitative study of physiotherapists and occupational therapists on two geriatric hospital wards in Norway that routinely tested their patients with standardized tests. Data draw on seven months of fieldwork, semi-structured interviews with eight physiotherapists and six occupational therapists (12 female, two male), as well as observations of 26 test situations. Data were analyzed using Systematic Text Condensation.

**Results:**

We identified two test information components in everyday use among physiotherapist and occupational therapist test administrators. While the primary component drew on the test administrators’ subjective observations during testing, the secondary component encompassed the communication of objective test results and test performance.

**Conclusions:**

The results of this study illustrate the overlap between objective and subjective data in everyday practice. In clinical practice, by way of the clinicians’ gaze on how the patient functions, the subjective and objective components of test information are merged, allowing individual characteristics to be noticed and made relevant as test performance justifications and as rationales in the overall communication of patient needs.

## Background

Standardized testing as a diagnostic activity in clinical settings is commonly thought of as a process involving three steps [1]. The first step is test selection, a step that has received some research attention even though it is not uncommon that medical institutions administer pre-chosen batteries of standardized tests to all suitable patients. The second step, which entails the administration and scoring of these tests, has also been researched although not to the same extent. The third step involves interpretation of test results. In this article, interpretation of results in relation to patients’ observed performance is the focus, as is the everyday use of test information which, we would argue, could be regarded as the fourth step of testing. By suggesting a fourth step, we want to draw attention to the fact that the physiotherapist (PT) and occupational therapist (OT) test administrators’ work does not end with the interpretation of test results. Test information, as addressed in this article, emerges in the form of scores and professional opinions that unavoidably build on test selection, test administration, and test interpretation. Test information is, thus, both a judgment and an outcome of processes of decision making [2].

By focusing on how test administrators in acute geriatric settings justify the use of test information in their everyday practice, this article investigates the complexities of everyday test information use, complexities that are particularly relevant when test administrators also are OTs and PTs who are responsible for parts of the patients’ health care. This focus is partly driven by the fact that test information can be used to determine level of impairment, disability, or activity since test information offers quantifiable documentation on patients’ functional ability. Test information can also be used to inform of and to monitor outcomes and, in some cases, to predict treatment outcomes [3]. Still, regardless of the multiple possibilities that test information offers, the clinical significance of using such information depends not on how it *can* be used but on how it *is* in fact used in a geriatric setting. The article focuses, therefore, on the latter.

### Using objective data while maintaining the clinician’s gaze

Whereas standardized testing has a long history in most medical specialties, non-standardized tests, informal interviews, and unstructured observation have been favored in OT and PT practice [4]. However, due to the implementation of evidence-based practice, a significant increase in the use of standardized tests is also noted in these two fields. This increase in testing activities is likely to continue, since health care professionals in general are under pressure to demonstrate clinical and cost effectiveness [5]. In any case, important to note is that the increase in standardized testing is not only about costs and effectiveness, but also about providing objective knowledge on patients’ health status [6,7]. Objective knowledge is often directly associated with quality and professional consistency in health care.

The traditional distinction between “objective” and “subjective” has roots in Cartesian philosophy, but it was the insights of Foucault in *The Birth of the Clinic*[8] that visualized the historic turn for objectivity in health care. Departing from the development of a diagnostic process built progressively on pathology, Foucault tells us of the medical doctors’ clinical “gaze” enabling them via pathological findings to see the patients objectively. Seeing objectively is here understood as being able to provide evidence on disease via observable and measurable changes in the physical body. Nowadays, objectivity is maintained through standardization; *“standards aim at making actions comparable over time and space; they are mobile and stable, and can be combined with other resources”* (p273) [9]. Not all standardized tests are diagnostic tools, but in health care, their main function is nonetheless to supplement the diagnostic process with an objective estimate of health status. Thornquist [7] notes, however, that PTs did not make a historic turn for objectivity, but upheld a clinical “gaze” that focuses on how the patient *functions*. The same functional orientation, though with an emphasis on daily living, is recognizable in OT practice and guidelines [10]. Thornquist [7] underscores that whereas the diagnostic process is recognized as being decontextualized, a therapist’s functional perspective takes the subjective experience, and the context, of the patient into consideration. This focus on patients’ subjective experiences, Thornquist argues, was not considered valid in the medical field because subjective experiences could not be measured and quantified. Almost ten years later, Sullivan explores what he calls a shift in current medical practice as he notes that patients’ subjective experience is called *“back into the center of clinical medicine”* (p1595) [6]. Worth noting in this regard is the fact that patients’ subjective experience has always had a strong foothold in geriatrics because preservation of functioning remains fundamental to successful treatment [11,12], but what is different in what Sullivan [6] refers to as *“the new subjective medicine”* is that patients’ subjective experience is quantified in objective health indicators. The medical notion of objectivity is thus being applied to enhance and legitimize subjective experience, such as the impact of functional ability on daily living and on quality of life measures. It is against this background of diverging professional traditions and expectations for data collection in health care that OTs and PTs are increasingly expected to collect and use quantifiable data.

Critical voices claim that without objective and systematic measures, professional health care is dependent on subjective skills and opinions – and, also, that the ability of clinicians to estimate functioning without such measures might be inadequate [13,14]. DeLuca and Putnam [15] deem the professional/technician model – i.e., the use of trained technical personnel to administer tests for health professionals – an efficient and cost-effective part of health care. More importantly, DeLuca and Putnam claim that the model allows for a more objective data collection, overcoming the potential for the professionals’ administration to be biased. Perhaps this is the case, but the professional/technician model does not address or solve the interactional challenges bound up in standardized administration.

Research into the interactional aspects of standardization has underscored that professionals and technicians alike experience a tension between what standardization demands and what individualization requires [16-25]. The result is often that the administrator departs from standardized administration. Any departure from standardized administration may affect results, results that, in turn, may limit the patients’ choice of, or access to, public services and treatment. It is these potentially dire consequences that Dingwall et al. refer to when they remind the reader that *“*[a test] *is only as good as what follows”*[18]. While this cause and effect argument is valid in most discussions on standardized testing, it is the tension in standardized administration set forth in these studies that represents the main challenge. We suggest that standardized administration should be understood in terms of its interactional characteristics rather than as an uninterrupted pathway to objective data.

Against the background of prior interactional work on the challenges of standardization, it seems appropriate to move our research focus to the everyday use of standardized test information and pose the following research questions: What information do the OT and PT test administrator collect from standardized tests? How do OT and PT test administrators use this information in their clinical work? While Tyson et al.’s [26] and Greenhalgh et al.’s [27] investigations targeted the uses of measurement tools and outcomes in multidisciplinary teams, we lack knowledge of how therapist test administrators use test information in their clinical work. To date, no studies have had access to hospital test situations and interviewed therapist test administrators to explore their justifications concerning the use of test information. This article addresses this research gap by exploring the everyday uses of test information from the perspectives of the OT and PT test administrators.

## Methods

### Fieldwork and participants

Data were collected on an acute geriatric ward and a stroke unit by the first author using fieldwork techniques. Observation and informal and formal interviews were undertaken over a seven-month period in 2009. A total of six OTs and eight PTs, two men and 12 women, participated in the study. They were from 22 to 54 years old and had from three months to 25 years of experience working with geriatric patients. Observations were made twice a week and organized so that the researcher spent one day a week on each ward following one of the 14 therapists around the ward in his or her daily activities. Approximately 170 hours were spent observing OTs and PTs work with nearly 90 geriatric patients, including observing 26 test situations.

Geriatric patients are generally associated with diminishing functional ability, reduced social network, and problems regarding the home situation [12]. OTs and PTs’ contributions are significant in the broad and multidisciplinary assessment geriatric patients need, and as part of their professional group’s responsibility in assessing patients’ functional abilities, they routinely conducted standardized tests. Most tests were delivered as part of a pre-chosen test battery, so in the 26 test situations, we observed close to 60 test administrations. Table 1 provides a summary of the standardized tests used in routine patient care. The test situations lasted about 30 minutes. Only patient, therapist, and researcher were present in the test situation, but on a few occasions, testing was conducted in a large training room where other patients and therapists were training. Short field notes were taken during observation and were expanded into more detailed descriptions at the end of the observation. The observational data are, in this article, used to contextualize and expand on the participants’ statements as they appeared in the interviews.

**Table 1:** Summary of standardized tests used in routine patient care

**Ward**	**Standardized test**	**Administrator**	**Description**	**Scoring**
**Acute geriatric**	Berg balance scale (BBS) [28]	PT	A test of 14 items to test balance and risk of falling in older adults.	A five-point scale, ranging from 0 to 4. Zero is lowest level and 4 the highest level of function. Total Score = 56.
	Timed “up and go” test (TUG) [29]	PT	Tests dynamic balance and mobility skills in older adults.	Timed in seconds. Lower than 10 seconds is normal. More than 10 seconds indicates reduced mobility.
	Mini-mental score examination (MMSE) [30]	OT	Samples cognitive functions such as arithmetic and recall ability, short-term memory, and orientation to time and place.	Full score is 30 points. Scores greater than or equal to 25 indicate normal cognition.
	Clock drawing test (CDT) [32]	OT	Screens cognitive and perceptual functions.	A modified version of Shulman [31] was used to rank clock drawings on a scale of 0 to 5, with 5 as best score and 0 as worst.
**Stroke unit**	Mini-mental score examination	OT		
	Clock drawing test	OT		
	Trail making test A and B (TMT) [33,34]	OT	Tests visual attention and task switching.	Timed in seconds. Higher scores reveal greater impairment.
	Motor assessment scale (MAS) [35]	PT	Tests motor function and muscle tone in stroke patients.	Each item is scored on a seven-point scale from 0 to 6.

The noted abbreviations will be used in presenting our findings.

Semi-structured interviews of approximately one hour’s duration were conducted in Norwegian with all 14 participants towards the end of the fieldwork period. For the purpose of this article, six key questions eliciting the participants’ perspectives on standardized testing were relevant. These questions were developed following long-term observation and tapped into contextual factors, professional judgment, issues of standardization, test feedback, as well as test utility. Except for one interview (in which the microphone batteries failed), all interviews were audiotaped and transcribed verbatim by secretarial staff. Quotes are translated by the first author and identified by profession (OT/PT) and by a number indicating the order in which the therapists were interviewed.

### Data analysis

In analyzing the interview transcripts, we used Systematic Text Condensation [36]. Systematic Text Condensation consists of four steps: (i) Independently read the transcripts to gain a contextualized impression of the interviews, and highlight preconceptions. (ii) Identify and code units of meaning – negotiate these until general agreement on the coding is achieved. (iii) Condense the meaning in the coded groups. (iv) Generalize descriptions reflecting therapists’ everyday use of standardized test information. Initially, we identified a series of smaller coded groups, each indicating a specific use of test information. However, as separate units these coded groups did not indicate how OTs and PTs actually oriented to test information. We then arranged the coded groups under the two summaries in order to indicate how patients’ subjective experiences were taken into account and, also, to indicate the role of test scores in communication.

Malterud [36] highlights the aspect of researchers’ preconceptions. In this study, the first author is a social anthropologist with no medical or health-related background. The second author is a sociologist and social gerontologist. The third author is a RN and the fourth author is a PT, both with clinical experience of working with older persons and their health care needs. The researchers’ different preconceptions of the geriatric context in general and of testing in particular proved to be valuable in interpreting the material. For instance, the fourth author has experience introducing and implementing standardized tests in PT practice and her preconceptions on the intention behind test implementation and understanding of test theory provided fruitful inputs in the interpretation process.

## Ethical considerations

The Regional Committee for Medical Research Ethics in Norway and the privacy protection ombudsman at the hospital gave ethical approval for the project. The therapists and other staff on the two wards were informed about the study in writing and verbally. Written informed consent was obtained from the 14 therapists and from all observed patients. The therapists recruited patients with ability to consent. No observation was undertaken until written consent was given. The PT and OT interviews commenced with verbal information about the study’s purpose and the participants’ right to withdraw, according to the Helsinki Declaration. All therapists received a copy of their transcript and were invited to comment. None commented.

## Results

The therapists interviewed are all expected by their institutions to administer standardized tests to all patients as part of their health assessment routines (Table 1). Test scores are entered into patients’ charts, and some test scores are also registered in hospital registers for research purposes. Overall, the administration of these tests was deemed to be time-consuming and some of the interviewed PTs and OTs stated that, at times, they felt that other rehabilitation-related activities were more important for the patients. This notion was strengthened by the fact that patients’ short stays at the wards seldom allowed for direct follow-up of test information. The findings that follow must be understood against the tension OT and PT test administrators experience in the test situation [16] as they navigate between the standardized procedures and the holistic orientation characteristic of best practice in geriatric patient care.

### The clinician’s gaze

OTs and PTs maintained that the test situation *per se* provided them with significant patient information. The test situation functioned as an arena for clinically observing the patient in action/interaction with the therapist. In addition to presenting the test’s stimuli (questions and tasks) and scoring the patient’s successive responses and performance, therapists explained that they would typically notice patients’ physical and cognitive functioning, coping strategies, emotional state, behavior, and ability to take instructions.

The therapists agreed that observing patients during testing provided them, as test administrators, with information on the patients’ functional status – a basic functional assessment:

PT2: (…) so, we observe basic functional ability: if they can sit, if they can stand, if they can walk, and if they can move about. That’s sort of what you observe in all (tests), also in BBS and TUG. (…). And something else that is common to be aware of is respiration. Then you’ll see … you’ll see how they breathe; heh-heh-heh (makes rapid breathing noises) high or if they do costal or abdominal breathing for example, or if they … because we often measure (oxygen) saturation on their finger. (…). Yes, (…) many need extra oxygen during activity. (Rows 541–549)

As implied in the quote above, the level of activity in physical testing was physically demanding for some patients. In fact, the level of physical activity in these tests was mentioned by several PTs as a beneficial by-product of testing, because the tests gave the patient a good workout. Thus, there was no need for the PT to treat the patient further on the test day. Another, and perhaps clinically more important, by-product of testing was that the functional ability of patients, observed while testing, could help therapists see what treatment measures the patient needed. Hence, observing patients’ impairments, such as potential respiration problems illustrated in the quote above, would trigger ideas for training schemes and aids needs. Another PT explained how observation of test performance was linked to training needs:

PT9: It gives me additional information, and it can also give me tips on what we should work with. (…). And you may see that he has troubles with the step (an elevated platform in BBS) and maybe we need to work a little more on that particular part of his balance, right? Or, I saw that the pace in TUG was much better when he used his walker than when he didn’t. So, that means that he’s able to increase his pace, but that he’s afraid to when he walks without support. (Rows 923–929)

This PT not only noticed what sort of balance training the patient needs, but also remarked the patient’s coping strategy, walking at a slower pace when walking without a walker. The therapists provided several similar examples of how patient strategies were observed in the test situation. The cognitive testing in MMSE offered an interesting example. The tenth question in MMSE is, “What floor of this building are you on?” Patients’ reasoning on this particular question was noticed:

OT10: Some are just so clever at this; “I arrived on the first floor and I cannot remember being wheeled up or down, no, I think I’ll go with the first floor.” And then, I consider them to be pretty clear-headed, but (of course, it is possible that upon admittance) they were placed in an elevator and just half-awake, and then you just don’t have a chance to keep track. (Rows 602–605)

Being attentive to patients’ strategies could also reveal their actual emotional state. Therapists remarked that some patients were insecure and scared upon entering the test situation, but that they played tough and defensive. This behavior was especially noticeable when testing cognitive abilities:

*OT12: (…) the ones that have experienced loss of memory and have had some a-ha moments where they’ve forgotten things – almost (started) a fire and things like that, they can be very like … refuse and not wanting to take it (the test). Because they’re scared that we’ll find out that it’s become worse. Some are acting very “but I know this.” If we ever get to (the MMSE question), “What country are you in?” (They’ll say), “What a stupid question, right?” (I’ll say) “Yes, can you answer it?” Because we need them to answer, and then you understand that OK here is* [the patient] *trying to hide something because the right answer isn’t coming. (Rows 568–575)*

Notice also how the therapist in this quote reasons about patients’ reluctance, but still justifies pressing for an answer.

Other test observations described by the therapists highlighted the patients’ physical behavior in test activities: Were patients fast or slow in their bodily movements? Examples of this were often visible in the physical testing; for example, the patient would finish the TUG quickly, but the therapist noticed that the patient almost fell several times during testing. In colleague communication, therapists often referred to such patients as “reckless”– not fully aware of their own physical limitations. Others were slow in their movements, and made sure they did not fall by walking slowly or checking that the chair was in the right position before sitting down. These patients were often referred to as “careful.” “Reckless” and “careful” indicated a mismatch between the patient’s capacity and behavior. Therapists also noted the cognitive aspect of patient behavior: for example, if the patient was adequate in conversation, or how well the patient comprehended test instructions.

Being a patient’s assigned therapist also entailed interaction (i.e., admission talk, training, and rehabilitation activities) with the patient outside the test situation. Therapists maintained that observations from outside the test situation often confirmed observations made in the test situation, but as one therapist pointed out, the opposite could also happen:

*PT13:* [Y]*ou turn away for a moment and suddenly they may be trying to grab a magazine lying on the table or another typical activity – and then suddenly their arm is as good as new. But when you are testing – oh, no then it’s not any good. But these things are kind of discovered because we see the patient during the whole day, right? (Rows 733–737)*

The OTs had an additional arena for observation because they habitually observed patients in morning care routines and kitchen safety training. These observations would typically serve as a backdrop for considering patient performance/behavior in the test situation.

### The economy of test score communication

Test scores are objective measures, but therapists seemed reluctant to accept that quantification was a particularly important aspect of their assessment. Instead, test scores were described as only providing a black and white statement, unable to capture all aspects needed in assessing geriatric patients and, thus, tests were not considered informative enough from the clinicians’ perspective. However, end scores still played a key role in everyday clinical communication.

Therapists claimed that standardized testing functioned as *“an assurance of quality of what we do, really. That it’s not just a discretionary, subjective assessment of things, but, like, doing a standardized test is maybe making it a bit more reliable too*” *(PT11 Rows 614–616).* In this quote, the notion of standardized tests as an objective base in professional statements is highlighted. It appears that, objective-based statements are considered to be better than subjective-based statements. And, although a few therapists argued that there must be a balance between subjective and objective statements, most therapists emphasized the test scores’ ability to support professional statements:

PT11: I feel that, in many ways, if we’ve done that test I’ve more weight in my argument when I call the district needs assessment office and order further physiotherapy (for the patient). Then I can, sort of, say that it isn’t just that the patient has reduced balance – that you’ve observed it, but you’ve also taken a standardized test which shows … (Rows 594–598)

To further underline the ambiguity surrounding objectivity and subjectivity, one therapist started out comparing test scores to results from blood tests and computed tomography (CT) to illustrate that test scores are, in fact, as objective as results from blood tests or CTs, but ended the quote pondering the professional dilemma that follows standardized testing:

*OT6: (…) they will take a blood test, they will take CTs of the head,* [but] *you will not see the cognitive impairments there. So, we need, sort of, something that can show that you do have cognitive impairments; that you have a problem conceptualizing time and then, the standardized tests are a good thing. (…) So, it’s somewhat the same thing, that these tests are important to provide the patient with the right treatment. At the same time, you cannot use them at random and you need to exercise professional judgment and be … understand that the patient is tired and sleepy – so, you need to consider that, and if the patient is unmotivated, then that may affect the result. (Rows 516–526)*

So, despite being aware of the possible limitations, and being somewhat critical towards quantifiable results from testing, therapists maintained that such results carry weight. The weight was in part linked to a medical system in which the quantifiable and objective were considered superior to the qualitative and subjective:

OT8: That’s always, sort of, been the good and the bad of medicine – that they’ve demanded numbers to ensure that something is true or not, right? And if you cannot quantify … things concerning quality of life and pain and such, then it’s harder to research it. But, the doctors are fond of everything that can be quantified, and what the doctors like propagates downwards in the system. That’s the way it is. (Rows 712–717)

But, weight was also given to the meaning inherent in end scores, as these described a specific level of functional ability. When therapists had experience with a particular test and its scoring system, they could define level of functional ability by score information only. One therapist highlighted this ability and exemplified how end scores, as opposed to a subjective statement on functional ability, left neither room nor need for interpretation:

PT11: (…) sometimes you may read an assessment where it says that the patient has reduced balance, but, OK, what is reduced balance? Does that mean that he, sometimes, needs to take an extra step when walking, or is he like really unsteady and walks, sort of, like a drunken sailor? That’s when it’s useful to have that number, saying that … yes, maybe it’s 45 points or it’s 5. (Referring to BBS scores. Rows 640–645)

Comparably, the therapists would look up earlier test scores on readmitted patients and compare them to new test scores. Two score sets illustrated the patients’ functional development by indicating progress, or lack thereof, over time.

This ability to understand scores was also emphasized as positive because it was knowledge most clinicians on the ward had in common: *“So, if you were to talk about a benefit then you’ve got shared understanding” (OT8 Rows 731–732).* In fact, it was the test scores’ position as objective and as a platform for shared professional understanding that made them function in communication with patients, colleagues, and districts’ needs assessment offices. A functional score may be used to assess patients’ needs for services and to allocate in-home aid equipment, placements in nursing homes, and other public health services in Norway. Thus, although we observed that OTs were somewhat reluctant to use scores in patient communication, in the interviews they stated that reluctance was mainly an issue if patients were frail or had low scores. PTs used test scores to communicate the age-appropriate function of patients or to illustrate fall risk. However, PTs communicated a score to patients with certain reservations well aware that:

PT1: It doesn’t mean anything to them, and I have to explain a little what it means. (…) Then I explain a little what the number means in relation to – in relation to the whole scale. And what the risk is, but then I’ll draw on … if I have seen the patient a lot I might know what the problem is.” (Refers to BBS. Rows 1104–1110).

Scores would be related to the patient in the following manner:

PT4: We talk a lot about the fact that “this test shows that you have a risk of falling and you have fallen, so this agrees well.” And we usually say something about the use of walking aids, and I say that “I see you’re good at using the walker and that you check that you sit down in the chair properly, because that’s what you need to do now. If you can (continue to) do that I’ll not worry.” (Rows 685–689)

As shown in the two quotes above, the quantifiable aspect of testing was not the main message to the patient. The few times therapists presented the end score as a main message seemed to be in communication with the district’s needs assessment office, because they knew that a low score could prompt allocation of public services. Still, therapists expressed reluctance toward this particular use of scores because it might entail testing patients who normally might be deemed unfit for testing:

OT8: I’ve had the district’s needs assessment office wanting MMSE to see if they can place the patient in a locked ward – and when you’re that impaired cognitively, then you’ll score down towards 15, 16. And then it’s a little … what’s the purpose of testing patients when we know that they’re pretty demented? (Rows 695–698)

Nevertheless, seeing that not all health care providers were familiar with tests’ scoring systems and that no end score could spell out the patient’s specific impairment, therapists habitually commented on the end score in writing: *“We never just write the end score in the chart. We always state what the problem is, because we are more concerned with the problem than with the actual end score” (OT6 Rows 514–516).* Also in verbal communication, for example, with the multidisciplinary team, end scores were likely to be commented upon:

*OT12: (…) it is important to me that you don’t say, in multidisciplinary meetings and reports, “27 of 30” and nothing more. You need to say what it is they scored poorly on and assess, that, yes,* [the patient] *was not oriented to place. (…). To me there is a difference between, like, you say one day wrong on date and day (questions) when you, like, are in a hospital and have been there for many weeks. Really, I’m not on top of dates and stuff every single day. You sort of need to consider this. But, if you say you’re in England when you’re in Norway, well, that’s a bit different. So, I think it is quite important to present what it was they scored poorly on, in order to get a more holistic impression of the patient. (Rows 443–453)*

A clarification of test scores, such as the clarification presented above, could help other health professionals localize and assess the clinical significance of a patient’s impairment. Testing benefitted from clarifications when therapists found that the end score did not approximate the real-life person *–* when there was a mismatch between observed behavior and end score.

OT14: (…) I had this patient who scored well on the MMSE, but when she was to brew a pot of coffee she didn’t have a clue how to do it. She didn’t understand why the water started to flow through and stuff. She’d turned the knob without noticing it. The same thing happened twice – and, like, according to the test score she should be pretty alert. (Rows 609–614)

Mismatches, such as this one, would typically be written down by the PT or OT as a caveat in the test form, communicated to the multidisciplinary team and, most likely, prompt further testing. Mismatches could, also, have an impact on how test results were communicated to the patient. For instance, if a patient scored high, but was considered reckless, the therapist would communicate the necessity of being more careful.

## Discussion

The tests delivered in this setting focused on loss of functional ability or on level of impairment, but since their administration is standardized, the results will not capture the individual characteristics of the patient [3]. Yet, this study’s findings suggest that individual patient characteristics are noticed and made relevant in the clinical use of test information.

### The two components of test information

The primary component of test information is gathered in the test situation, where it is apparent that therapists are not only test administrators; they are also observers. The therapists see the individual they test; they see their patients. During testing, they take in the patient’s physical and cognitive functioning, emotional state, coping strategies, conduct, and ability to take instructions. In fact, these observations are, in the therapists’ accounts, often presented as the therapists’ key concerns and they can be used to support or challenge decisions regarding patients’ forthcoming activity and treatment plan: the patient is sad, the patient needs to use a walker, or the patient is slow/fast and careful/reckless. Such concerns and typologies resonate with Thornquist’s [7] portrayal of therapists as attentive to patients’ subjective experiences and to their functional abilities. On the other hand, concerns, such as the ones presented here, may also influence the clinicians’ ability to score their own patients accurately [37,38]. It may be the therapists’ twin position, as test administrators and as the particular patient’s therapists, that makes them attuned to collecting information that extends beyond what standardized testing deems significant. One example of therapists’ collecting information that extends beyond the standard is provided when therapists note patients’ coping strategies; another example is provided by the therapist who claimed patients’ malingering in tests was discovered *“because we see the patient during the whole day” (PT13).* If tests were delivered by a technician, as suggested by DeLuca and Putnam [15], this information would likely be lost. In actual fact, the therapists’ broad approach to test data suggests that they do not heed the underlying distinction between testing and assessments; the subjective component present in health assessments should be absent in standardized testing [39].

The secondary component of information falls, principally, in the category of quantifiable test data: end scores. Scores and end scores provide the health care professional with quantifiable documentation on patients’ *status quo* functional ability. Insights on how therapists use quantifiable data can be summed up by Fujiura and Rutkowski-Kmitta’s statement: *“Numerical associations facilitate independent verification, standardization, and economy of communication”* (p92) [40]. There were no independent verification procedures in this clinical setting, because no disinterested third party was involved in test interpretation. However, involvement by interested third parties could occur when therapists discussed observations and test scores among themselves or in the multidisciplinary team. In addition, therapists expressed a notion of trust in standardized tools as objective. They compared, for instance, findings from standardized tests to pathological manifestations visible in blood samples and CT scans. Trust in standardization was also demonstrated when scores from previous hospital stays were compared to the patient’s new scores.

### Understanding the numbers

The two components presented in our study find a parallel in Polanyi’s [41] distinction between tacit and explicit knowledge. Tacit knowledge is subjective and created through direct experience [42]. Tacit knowledge, thus, embraces an array of conceptual and sensory information and images (*we know more than we can tell* (p4) [41]), whereas explicit knowledge is the knowledge we are able to articulate, standardize, codify, and store. In line with Greenhalgh et al. [27], our study brings to the fore the interaction between tacit and explicit knowledge in the use of test information. The guiding role of subjectivity in the therapists’ use of “objective” information illustrates how “facts,” such as test scores, do not speak for themselves, but instead are interpreted and translated [2,43]. Thus, information is not given or is not *“the outcome of individual minds, operating in a social vacuum”* (p54) [2]; rather it is the result of a continuous collective interactional activity that produces, interprets, and translates it from one setting to another.

At the outset, numbers are considered objective, and in the therapists’ accounts, objective data, that is, numerical data, are associated with quality, reliability, and credibility. A similar association between objective data, reliability, and quality is noticeable in the rhetoric surrounding continuous upgrading of health care provision (see, for instance, [5,6,44]). Still, with regard to the expressed credibility of objective data found in our material, we noted ambivalence among participants: Objective data, numerical data, were often depicted as mere black and white and of limited use or value to clinicians – simultaneously, scores were frequently used in communication. This brings us to a main finding regarding the secondary component of information, a finding that concerns what Fujiura and Rutkowski-Kmitta label “*the economy of communication.”* The economy of communication on the wards studied here is seemingly sustained in a multifaceted communication practice that, in fact, goes beyond numerical representation. Thus, we argue that to the therapist familiar with the specific standardized test, the score numbers contain information that goes beyond mere numerical representation. Test scores state level of impairment, often in relation to a normative sample and are, as such, encoded [42]. Knowledge of a test’s scoring system and its normative sample is necessary in recognizing the level of impairment indicated by the end score [1]. Atkinson [2] describes information or encoded knowledge as embodied in different forms of representation (test scores, laboratory test results, MR printouts). His perspective underlines not only that tacit knowledge is key to the production of scores, but also that it is key in generating and maintaining the scores as explicit knowledge. Although encoded knowledge does not preserve the tacit skills of the individuals generating it [42], it provides the therapists with a common language, essentially a shared understanding, of scores. This common understanding facilitated communication with colleagues, as well as communication with the districts’ needs assessment offices, but it seemed to fail in communication with patients. Patients, as opposed to colleagues, had no understanding of the message in numbers, and therefore had to have them explained. Therefore, in providing test feedback to patients, the primary component of information was used as the main information source. In practice, the therapist would communicate a contextualized image of a decontextualized test to the patients [16,45].

The ambivalence noted among the participants regarding numerical representation was not directed at the scores’ inability to provide insight into level of impairment; it was directed at the scores’ inability to capture patient’s characteristics [3]. Our analysis shows that, in line with research conducted from an interactional perspective, patients’ characteristics and the context are relevant in face-to-face standardization. In interactional-oriented research, test administrator characteristics, patient characteristics, wording, and context have been shown to affect test results. This study, however, suggests that only patient characteristics and context are made relevant when test administrators justify their use of test information. The fact that test observations routinely were made known in the form of written caveats illustrates the therapists’ wish to contextualize patient performance. In practice, caveats render visible tacit knowledge in standardized outcome measures: the manner in which clinicians’ intuitive judgment, reasoning, and expertise are used to supplement, dismiss, or adjust scores [27]. Thus, caveats highlight what the end score could not: the patient’s problem – *“we are more concerned with the problem than with the actual end score” (OT6).* This practice also underlines the therapists’ pragmatic stance towards testing. A similar approach to test interpretation is found in Dingwall et al. [18]. Caveats were especially important when a mismatch between patients’ observed behavior and the end score was noted. Therapists’ uses of caveats provide an example of how:

*External clinical evidence can inform, but can never replace, individual clinical expertise, and it is this expertise that decides whether the external evidence applies to the individual patient at all and, if so, how it should be integrated into a clinical decision.* (p71) [44]

The objective contribution of standardized tests proved to be moderated by caveats. Caveats were actively used in seeking agreement between the subjective and objective components of test information. Therefore, therapists challenge the sole use of one of the components.

### Limitations and further research

Although the focus of this article has been on professionals’ test information use, important issues possibly affecting their everyday use of such information are left unexplored. First, the health professional’s work experience is likely to influence how results are interpreted and, also, what test observations are deemed relevant in planning rehabilitation and communicating patient performance. Second, two wards and two professional groups were studied, but we did not explore the potential differences between test information use on the wards or between the two groups of professionals. Taken together, these issues could help provide a fuller picture of standardized testing. In addition, we suggest that the use of caveats should be investigated further. Yet, to fully contrast our findings, we recommend research into health care settings where end scores are delivered by a technician.

## Conclusions

We stand a better chance of understanding the complexities of everyday use of test information in this particular setting if we take into account the twin position of the therapist, as the patient’s OT or PT and as test administrator. Our findings suggests that, in clinical practice, by way of the clinicians’ gaze on how the patient functions, two different components of test information are merged, and that in the overlapping of these components, individual characteristics are made relevant as test performance justifications and as rationales in the overall communication of patient needs. The overlapping of subjective and objective test information should be investigated further to make known the implications the clinical use of test information may have on the provision of health care.

## Competing interests

The authors declare no conflict of interest with regard to the authorship and/or publication of this article.

## Authors’ contributions

ÅS and AB conceived of the study and KK developed its methodology. KK conducted fieldwork and interviews. KK, ST, ÅS, and AB contributed to the interpretation of the findings. KK drafted and wrote the manuscript. All authors commented on different versions of the manuscript, and read and approved the final manuscript.

## Pre-publication history

The pre-publication history for this paper can be accessed here:

http://www.biomedcentral.com/1472-6963/14/72/prepub
